# An economic analysis of the limits of market based reforms in the English NHS

**DOI:** 10.1186/1472-6963-13-S1-S1

**Published:** 2013-05-24

**Authors:** Pauline Allen

**Affiliations:** 1 London School of Hygiene and Tropical Medicine, London, UK

## Abstract

**Background:**

Over the past three decades, a limited range of market like mechanisms have been introduced into the hierarchically structured English National Health Service (‘NHS’), which is a nationally tax funded, budget limited healthcare system, with access to care for all, producing structures known as a *quasi* market. Recently, the Health and Social Care Act 2012 (‘HSCA’) has been enacted, introducing further market elements. The paper examines the theory and effects of these market mechanisms.

**Methods:**

Using neo-classical economics as a primary theoretical framework, as well as new institutional economics and socio-legal theory, the paper first examines the fundamental elements of markets, comparing these with the operation of authority and resource allocation employed in hierarchical structures. Second, the paper examines the application of market concepts to the delivery of healthcare, drawing out the problems which economic and socio-legal theories predict are likely to be encountered. Third, the paper discusses the research evidence concerning the operation of the *quasi* market in the English NHS. This evidence is provided by research conducted in the UK which uses economic and socio-legal logic to investigate the operation of the economic aspects of the NHS *quasi* market. Fourth, the paper provides an analysis of the salient elements of the *quasi* market regime amended by the HSCA 2012.

**Results:**

It is not possible to construct a market conforming to classical economic principles in respect of healthcare. Moreover, it is not desirable to do so, as goals which markets cannot deliver (such as fairness of access) are crucial in England. Most of the evidence shows that the *quasi* market mechanisms used in the English NHS do not appear to be effective either. This finding should be seen in the light of the fact that the operation of these mechanisms has been significantly affected by the national political (i.e. continuingly hierarchical) and budgetary context in which they are operating.

**Conclusion:**

The organisational structures of a hierarchy are more appropriate for the delivery of healthcare in the English NHS.

## Introduction

Although England in the 20^th^ and 21^st^ century is generally a market based, capitalist society, as far as the production of most goods and services is concerned, it is notable that the funding and delivery of many public services has not been left entirely to markets. Nevertheless, in the last few decades, there has been a notable increase in the use of market mechanisms in public services including healthcare [1]. This paper will examine the theory, possible difficulties and actual effects of introducing market like mechanisms into the hierarchically structured English National Health Service (NHS), bearing in mind that the NHS is a nationally tax funded, budget limited system, with access to care for all. I will argue that there are serious limits to the efficacy of using economic market principles in the delivery of healthcare. (The paper does not seek to provide a historical, political or political economy analysis of *why* markets have become more prevalent in English public services. It provides a technical analysis based on economic theory and evidence.) The paper consists of a first section examining the fundamental elements of markets, comparing them with the operation of the mechanisms of authority and resource allocation employed in hierarchical structures to deliver public services. The second section discusses the application of market concepts to the delivery of a complex product such as healthcare, and draws out the problems which economic and socio-legal theories predict are likely to be encountered when attempting to use markets in healthcare, and discuss the theoretical reasons for the use of hierarchies to deliver public services instead. The third section then discusses the evidence concerning the actual operation of market elements in the NHS. The fourth section discusses the future operation of market instruments under the regime set out in the Health and Social Care Act 2012, and the conclusion will reiterate the case for hierarchical governance of NHS services in England.

## Theory

### Economic theory of markets

#### Basic economic theory of the perfect market

In order to understand the logic behind introducing market mechanisms into public services, it is necessary to understand how perfect markets are meant to work, and what their advantages over other forms of institutional structure are thought to be. A brief and simplified explanation of the key concepts of markets follows. The core notion is that there are willing and rational buyers of goods or services (demand) and willing and rational providers of goods or services (supply). In order for a market to operate efficiently, there need to be sufficient numbers of each for competition to occur [2]. Economic theory states that, in these circumstances, the price at which goods/services are purchased will be the most efficient one (the equilibrium). In pure markets, demand is expressed by the individuals who receive the goods/services making choices themselves and paying their own money. It is assumed that consumers are able to find and process sufficient information about the goods/services to make rational choices. These conditions ensure that each person’s individual utility is maximised, as they each are the best judge of what will achieve this. Thus, the market has produced value for money by allocating resources to the best use. The mechanism by which demand and supply is brought together is a contract, under which the parties are free to agree whatever terms they wish, and which will be enforced by the legal system. In perfect markets, not only is efficiency achieved, but so is accountability, as each consumer has made her own decision based on adequate information, and the terms of the agreement between the consumer and supplier can be enforced against the supplier using contract law.

#### Lack of perfect market leads to need for regulation

It is recognised that markets for many (if not most) goods and services do not accord with these principles, so adjustments are made. It is often the case that a market will not be perfectly competitive, due to distortions on the supply side (including natural monopolies caused by the nature of the product) and/or asymmetries of information between supply and demand, and regulation will be required in order to ensure that suppliers do not use their market power to increase prices above the efficient level, or to provide low quality products [3]. In theory, if regulation is performed appropriately, most of the advantages of the market can be attained.

#### Markets not concerned with equity

It should be noted that theories of markets do not classically concern themselves with problems in the fairness of the distribution of goods and services [4]. Consumers simply use the resources they have available to purchase what they most desire, thereby maximising their individual utilities. The notion of needing or deserving services which cannot be purchased is not included.

#### Limitations to economic theory of markets

Of course, this simple economic theory of markets has been subject to many criticisms over the years (e.g. [5,6]). The most important ones are a) that it is wrong to assume that human motivation is primarily self-interested [5]; and b) that this type of approach ignores the fact that economic transactions are embedded in social relationships [7-9]. This paper will not deal with these important points – rather, it seeks to use an economic framework to analyse and question the effectiveness of markets for healthcare.

### Theory of hierarchies

In contrast to markets, where power is distributed among all participants, in a hierarchy there is a single person or group at the top with the most power and authority, and each lower level represents a lesser authority. Instead of competition between freely operating providers, decisions about supply are made inside the hierarchy using authority. Consumers do not make direct consumption decisions either: the hierarchy allocates goods and services to them. Many public services are organised according to these basic principles. Instead of direct payment for services, governments collect taxes and use these resources to produce services and allocate them to consumers. One of the reasons for this is that notions of rights and need are thought to be important determinants of allocation of public services [10], and hierarchies are needed to make decisions on behalf of collectivities of people, taking account of these concepts.

Classic economic theory sees hierarchical structures as relatively inefficient compared to markets. This is because centralised authority is employed to make decisions about the use of resources, rather than the ‘invisible hand’ [11] of the market, under which many individual decisions are aggregated to form an equilibrium. The former may reduce efficiency in production of goods/services. Furthermore, Williamson [12] notes that the lack of strong incentives in hierarchies, compared to contractual relationships in markets, is likely to reduce the efficiency of command and control structures.

In addition to possible reductions in efficiency, hierarchies introduce additional questions about accountability to consumers of services which do not occur in pure markets. It is primarily decisions on the demand side which become more complex as decisions are not made by each consumer, but by someone else in the hierarchy on their behalf. In these circumstances, the notion of holding someone to account for those decisions becomes meaningful. A useful definition of accountability is ‘a relationship between an actor and a forum, in which the actor has an obligation to explain and to justify his or her conduct, the forum can pose questions and pass judgement and the actor may face consequences’ [13]. There are various ways in which these decision makers can be held to account including (depending on the circumstances) use of legal sanctions, locally agreed contracts and public elections. All of these have their problems [14-16]. It should be noted that there are intermediate forms of organisation between markets and hierarchies, which are often labelled ‘networks’ (e.g. [17]). For the sake of clarity, discussion of these is not included in this paper, whose focus is a critique of the use of markets in the NHS, not a general survey of possible organisational forms for the delivery of healthcare. Hierarchies have been presented here as the alternative to markets as they are theoretically the most useful contrast to markets; and also the prominent existing (and historic) organisational logic in the NHS.

### Limits of markets in healthcare and other public services

#### Use of hierarchies in public services

Despite the fact that simple economic theory indicates that markets will be more efficient than forms of hierarchy and accountability will not be so complex, we see that many public services have been delivered through institutional structures which more closely resemble hierarchies than markets. This is because the basic principles of market theory do not apply to many public services, and particularly not to healthcare [4].

A very brief explanation of why this is the case follows. Firstly, concerns about need and entitlement immediately intrude – in England, we take the view that people are entitled to healthcare, even if they cannot afford to buy it when they need it. As the market for insurance against healthcare expenses does not work properly [4], in England we choose to fund healthcare using compulsory taxes. This means that consumers are not using their own resources to make decisions about healthcare use. Nor are they usually making choices about their provider, as this is made on their behalf by government officials acting as agents. Secondly, the complex nature of healthcare means that patients find it very difficult to obtain and digest sufficient information to make their own decisions about their consumption – professional expertise and advice is the most important aspect of healthcare [18]. There are significant asymmetries of information between providers of care and patients. (And, indeed between agents acting on behalf of patients.) This means that the market condition of perfect information does not apply. Thirdly, due to the complexity of healthcare, it has to be regulated to ensure quality is maintained. This means that there will not be total freedom of supply. Moreover, there are also large costs associated with establishing and running hospitals, there may not be sufficient suppliers to ensure competition occurs [4]. Lack of full competition, together with the other missing factors, will affect the capacity of a market for healthcare to be efficient in the sense of maximising individual utilities.

#### Theory of the quasi market for healthcare

Due to these factors, the form of market for healthcare introduced into the hierarchical system in England was in fact a quasi-market [19]. This sought to combine the advantages of competition between suppliers with the safety of retaining public funding to protect fairness in access to care [1]. An internal market for community, secondary and tertiary health care was introduced by means of a split between the purchasers of care (health authorities, which were local outposts of the NHS state hierarchy, and GP fundholders, who were some general practice physicians who chose to hold a budget of NHS money to spend on certain aspects of NHS care for their patients) and its providers. The providers of health care were constituted into relatively autonomous ‘self governing NHS Trusts’ (still publicly owned), who were supposed to compete with each other, thereby enhancing technical efficiency (that is ensuring the greatest output for the least resources used, i.e. ‘value for money’) [19]. Proponents (such as Enthoven, [20]) contended that technical efficiency was more likely to be achieved in a situation of competition between providers, which could make their own decisions about modes of delivery of care, than in a structure (such as a hierarchy) which effectively contained monopoly providers. Decisions about consumption of community, secondary and tertiary health care were made by agents in the state hierarchy on behalf of patients [21]. (There have since been changes in this aspect of the quasi market to increase individual patient choice [22]. These will be discussed below.) The system of annual budget allocations was to be replaced with one based on negotiated contracts between purchasers and providers [19].

**Figure 1:**
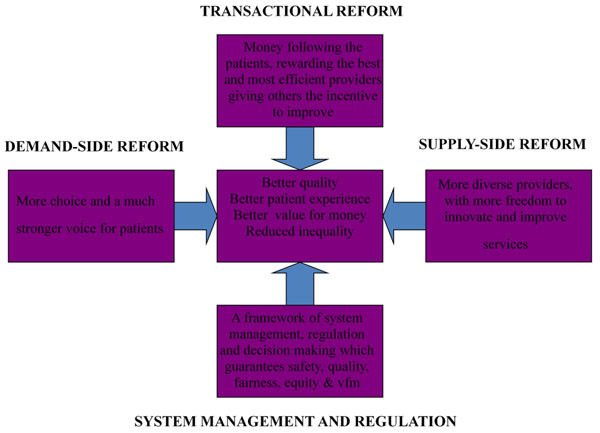
The four interrelated pillars of reform.

A Department of Health (i.e. the national Ministry of Health in England) diagram of the quasi market under the New Labour government demonstrates how policy makers envisaged that the different economic building blocks would work together to improve efficiency and quality of care. See Figure 1. The system attempted to bring together the necessary quasi-market elements I have discussed above in a manner which sharpened the market incentives compared to the Conservatives’ quasi market of the 1990s. The emphasis on quasi-markets as a motor for improvement is encapsulated in ‘four inter-related pillars of reform’ which ‘are designed to embed incentives for continuous and self sustaining improvement’ and produce ‘better quality, better patient experience, better value for money and reduced inequality’ [23]. These are: ‘(i) Demand side reform - more choice and a stronger voice for patients; (ii) Transactional reform - money following patients, rewarding the best and most efficient providers, giving others the incentive to improve; (iii) System management and regulation - a framework of system management, regulation and decision making which guarantees safety and quality, fairness and equity; and (iv) Supply side reform – more diverse providers, with more freedom to innovate and improve services.’ [23].

#### Theoretical limits to the quasi market: related economic and socio legal approaches

While a quasi market has been seen by some as the best solution to providing efficient and high quality healthcare [1,20], aspects of economic and legal theory indicate that serious problems could be encountered in moving from the pre-existing hierarchy to such a system.

The first issue is that of demand side and agency. As patients are not able to make their own consumption decisions, the demand side of the quasi market still relies on parts of the state (and GPs) making decisions for them. In order for the market to work, these agents would have to make decisions only taking into account patients’ goals, Agency theory [24] indicates that it will be very difficult to align their goals to those of patients. Propper [21] explains that the fact that health care is publicly funded means that the government needs to monitor its agents. But monitoring is problematic: in the absence of perfect information, the principal will not be able to tell whether the agent has failed to perform or, if there is a failure to produce the required services, whether the agent was responsible for that failure (by shirking, for example) or whether it is due to unforeseen circumstances [25]. Propper [21] also points out that it is clear that the government will not be able to monitor all aspects of output and that partial monitoring is likely to reduce the incentives on agents to undertake other dimensions of performance which are not being monitored. The introduction of patient choice is partly aimed at dealing with this problem. But it cannot obviate it, as the nature of healthcare means that patients are not able to act as fully informed, rational consumers.

The second issue concerns the supply side. The nature of healthcare means that there is unlikely to be a large degree of competition, especially in respect of specialist hospitals, and in sparsely populated areas [4]. These two issues are likely to decrease the possibility of overall efficiency being improved by using a quasi market.

The third issue concerns the central mechanisms of the quasi market: pricing and contracting for care. As it is impossible to monitor all aspects of healthcare performance, economic theory predicts that competition involving negotiated prices would reduce quality [26], as providers can skimp on unobserved aspects in order to lower their costs [25]. In order to avoid this problem, fixed prices were introduced gradually into the NHS quasi-market from 2003/4, so that competition should be based on quality alone [27]. However, this does not obviate the problem that not all quality can be observed, and skimping on quality and intensity of treatment may occur [28]. Moreover, the price has to be fixed somehow, and this process may cause other difficulties. If the price is too low, although providers may be encouraged to become more efficient, they still may be able to skimp on quality. If the price is too high, there is no incentive for providers to become more efficient [29].

The problem is that it is not possible to monitor all aspects of health care affects more than price setting, in fact. It has the potential to undermine the effective use of contracts in healthcare markets, both in terms of increasing efficiency and in achieving accountability. The literature on the economics of contracting indicates that the transactions cost of contracting for healthcare will be significant. If these are taken into account, the supposed increased efficiency produced by competition in a market system is made more questionable. When contracting for a complex service, such as healthcare, a range of contractual difficulties are likely to occur. Briefly, these are difficulties in specifying what is required, and problems in monitoring caused by asymmetry of information and the complexity of services and also the ever present possibility of opportunism [12]. Transactions costs result from imperfect information, either about the other party involved in the exchange (asymmetric information) or about the future (uncertainty). Imperfect information means that it is costly to enter into contracts for the exchange of rights, since the parties will have to incur the costs of searching for a suitable trading partner and then negotiate and write contracts (*ex ante* transactions costs). It also makes it costly to monitor, enforce and renegotiate contracts (*ex post* transactions costs). Both types of transactions cost may be high, but when there is a high level of uncertainty about relevant events there may be a trade-off between the two: the costs of making contracts may be reduced by not attempting fully to specify contingencies, leaving a contract incomplete and necessitating renegotiation (leading to *ex post* transactions costs) to accommodate events left out of the contract. This means that such contracts will not be ‘complete’ [12].

Complementing the economic approach, an influential strand of socio-legal contract theory focuses on the way in which transactions are underpinned by a combination of *discrete* norms, such as consent and choice, that are necessary for planning and presentation (i.e. the deliberate attempt, through planning, to bring the future into the present), together with *relational* norms such as flexibility and reciprocity that are essential to support trust and cooperation [30,31]. Contractual exchanges are said to be ‘relational’ to the extent that they reflect an appropriate balance between discrete and relational norms, thereby creating the conditions for the attainment of the joint welfare-maximising benefits associated with successful contractual relationships in business practice. (Successful contractual relationships in business practice allow both parties to achieve the goals they regard as most important. These might include, from the seller’s point of view, selling goods at a fair price which allows a profit to be made; and, from the buyer’s point of view, obtaining goods of acceptable quality at a fair price.) The combination of contract norms varies according to the nature of the transaction, with the discrete norms being particularly prominent in short-term or spot exchanges, while relational norms are more evident in continuing or long-term contractual relationships. This combined economic/socio-legal theoretical perspective predicts that the quality of relationality, while a necessary precondition of the success of all contractual relations, may be difficult to achieve in market exchanges where the characteristics engendering high transactions costs pertain [32]. This means that, despite the views of some commentators [33], trust and cooperation will not be able to make up for deficiencies in the contract in respect of specification and monitoring.

Finally, the very difficulties which lead to high transactions costs in healthcare contracting also indicate that it will be difficult to use these contracts to achieve full accountability from providers of care, as it will not be possible to monitor all aspects of performance. This means that the simple model of markets as a method to improve accountability is flawed, as it does not take account of the difficulties in contracting.

#### Theoretical advantages of hierarchies for healthcare delivery

The foregoing considerations indicate that there may be some advantages in retaining hierarchies as the institutional structure for organising healthcare. For example, transactions costs theory indicates that hierarchies may be more efficient in the provision of healthcare. The stronger incentives of markets may be detrimental to efficiency and quality of care where those incentives cannot be effectively harnessed for the public good. Once the incentives of markets are introduced, one can expect stakeholders to avail themselves of the opportunities they present, and the stronger the incentives, the more likely they are to change behaviour. For example, if for profit providers of care enter the market, these firms have stronger incentives to skimp on quality to increase their surpluses, as these can be distributed as profit. In addition to the economic reasons, as mentioned earlier, one very important reason why governmental hierarchies are often preferred for the delivery of public services is that considerations of fairness of access and additional social value (in the form of externalities) are equally important to policy makers as efficiency [34]. These functions are better handled by the exercise of authority in command and control structures than in the decentralised decision making of individuals in markets.

### Evidence on the operation of the quasi-market in the English NHS

In this section, I will provide a brief overview of the evidence concerning the operation of the NHS quasi-market, using the theoretically based topics about markets in healthcare outlined above to structure the analysis. (Fuller accounts of the effects of the quasi market policies of the two successive governments of the last twenty five years (ie. the Conservatives and New Labour) respectively can be found in [35] and [36].) The evidence cited here is derived from research conducted by researchers based in the UK which uses economic and socio-legal logic to investigate the operation of the economic aspects of the NHS internal market using its own theoretical basis. It does not seek to include wider literature which criticises the logic of markets generally, nor in the NHS specifically e.g.[37]. Moreover, space constraints do not allow me to include the extensive discussions of the methodological approaches used in recent studies of quality and competition (e.g.[38] and [39]).

#### Demand side of the quasi-market

Starting with the demand side, research has indeed demonstrated that agents acting on behalf of patients have not been effective [21,35,40-42]. These agents hardly used their market power to make changes. Moreover, the more recent introduction of a policy allowing a degree of individual patient choice of provider has not been widely taken up in practice, and does not seem to have had an appreciable effect on providers [43,44]. One reason for this is likely to be that patients are not willing or able to obtain and process the necessary information, as theory predicts. An important aspect of the demand side relates to the operation of the quasi market as a whole. This is the role of state organisations which commission care on behalf of patients (latterly these were called primary care trusts, PCTs) in relation to market entry by non NHS owned providers. It appears that the attitudes of PCTs have varied considerably, and that their behaviour can make the difference between such providers gaining a foothold in the NHS or failing to do so [45].

#### Supply side of the quasi-market

Evidence about the supply side in English quasi markets shows that there has not been a large amount of competition between suppliers [46] and [47]. As economic theory predicts, where competition occurred under negotiated prices, quality of care reduced [46]. On the other hand, there is some emerging evidence that competition under fixed prices may have increased quality of care [47]; and [48], as economic theory predicts.

The other aspect of the supply side which is thought important in economic theory is the degree to which organisations providing care are free to make their own decisions, as opposed to being controlled by, for example, a government hierarchy. This freedom is thought to be important in achieving more efficient and higher quality care, as those actually running the organisations have better information about how to organise delivery to respond to patient need and demand [49]. Evidence from research about more autonomous NHS (i.e. state owned) hospitals introduced by New Labour (Foundation Trusts, FTs) does not bear this out because those hospitals selected to become FTs were already performing better than their comparators –thus it cannot be inferred that FT status was the cause of better performance [50]. The research evidence also indicates that FT autonomy has been severely circumscribed, and the national NHS hierarchy still has a strong influence on FT decision making [51] . Moreover, there is no evidence to suggest that independent providers (whether for profit or otherwise) are performing better (or indeed, worse) than NHS owned organisations. It should be noted, however, that very little evidence is actually available to date, and none in respect of efficiency [50].

#### Economic regulation of the quasi-market

Economic theory also predicts that regulation will be a key instrument in improving the workings of the NHS quasi market being designed to encourage competition and deter monopolies [4]. There does not appear, however, to be any research evidence concerning the economic regulation of the NHS quasi-market [52]. The current competition regulator is the Cooperation and Competition Panel (CCP), which is a government body established at arm’s length from the Department of Health. The CCP’s mandate demonstrates that it has a series of concerns to take into account simultaneously, not all of which deal with promotion of competition. It has to follow the Principles and Rules of Co-operation and Competition set out by government [53]. These state that both co-operation *and* competition are desirable . Furthermore, its actual decisions demonstrate that considerations other than promotion of competition have been taken into account [54].

#### Pricing and contracting in the quasi-market

Turning to the key mechanisms of the NHS quasi market – pricing and contracting – we find that available evidence bears out the issues raised by theory in some respects, but not in others. Research about the use of the national tariff (PbR) indicates that there is no evidence to support theoretically based concerns about it incentivising skimping on quality [28]. Difficulties in setting the correct prices have been encountered (as expected) and it is not clear what effect the tariff has had on hospital efficiency [28]. It should also be noted that the national tariff has not in fact been used on all occasions when it has been mandated for use. The NHS hierarchy has stepped in to blunt the effects of this payment by activity system, as commissioners did not have sufficient funds [32,55,56].

As far as contracting is concerned, the evidence has confirmed the difficulties raised by transactions costs and socio-legal theory. It has not been possible to make complete contracts for healthcare, specifying and monitoring quality has proved problematic, and thus, contracts have not been able to achieve full accountability of providers [32,42,57,58]. Although a high degree of relationality has often been achieved, the development of a measure of trust and cooperation in some contractual relationships has not been able to make up for lack of completeness. The introduction of a national standard contract in 2007 with the aims of obviating duplicated effort on the part of commissioners, and of improving the detail of specification and monitoring has not significantly improved matters [32].

#### Continuing use of hierarchy in the quasi-market

In addition to considering evidence about the operation of the economic building blocks of the NHS quasi market, it is necessary to bear in mind the degree to which command and control hierarchical measures continue to operate concurrently [20,35,36]. Some of these hierarchical measures have been mentioned in the discussion of the evidence above. One of the reasons why these measures continue, despite an increasingly enthusiastic espousal of market principles by the New Labour government during its term of office from 1997 to 2010 [59], is that policy makers have a range of goals for the NHS, not all of which can be met by using market mechanisms. These additional goals include promoting continuity of care for patients (which requires cooperation between providers – hence the need for the CCP to include principles on cooperation, as well as competition); meeting national standards for quality; and keeping the whole NHS within nationally set financial limits. As Jessop [60] points out, the Third Way State undertakes ‘meta-governance’ by a ‘judicious mixing of market, hierarchies and networks’. In New Labour’s NHS, these hierarchical policies included most notably the continuing use of national targets for issues such as reducing hospital acquired infections and reducing inpatient waiting times [61]. Introduction of a standard form of contract to be used in the market regime somewhat undermined the notion of a market as involving the devolution of power. Finally, each year an operating framework was issued to the NHS by the Department of Health setting out annual priorities for the whole system to follow (e.g. [61]). Not only did these blunt the efficacy of financial incentives, but they also overrode demand side *and* provider autonomy to some extent [51].

### Quasi-market policies of the Coalition government

This paper has shown how economic market principles are difficult to apply to healthcare, and some of the consequent challenges which the NHS has faced in operating a quasi-market for the last two decades. The Coalition government has made further policy changes to the NHS in the Health and Social Care Act 2012 (HSCA), which is a major piece of national legislation dealing with the structure of the NHS in England. These mainly continue in the direction that the New Labour government set in its later reforms [62]. In addition to the changes on the demand side (which are not germane to the economic argument, as patients will continue to have a mixture of agents acting on their behalf and some degree of individual choice), there has been a serious attempt to introduce full economic regulation. Monitor will take over as economic regulator, and there will be a statutory obligation on it to promote competition (HSCA, s 62 (3)), and it will be obliged to carry out anti competitive regulatory functions in conjunction with the Office of Fair Trading under the Competition Act 1998 (HSCA s 72). (Monitor is a government body originally established at arm’s length to the Department of Health to authorise and regulate the activities of FTs.) Nevertheless, it should be noted that the enacted version of the HSCA also requires Monitor to take into account a wide range of other goals, including integrating services (s 62 (4)); ensuring fair access to care; improving the quality of care; and obtaining value for money (HSCA s66)*.* The force of economic regulation to promote competition has been significantly blunted by the additional provisions in the HSCA (and thus pure economic market principles have been compromised). The detailed regulatory and licensing provisions of the HCSA are also designed to promote the entry of independent providers of care into the NHS quasi market by ensuring there is ‘level playing field’ for these organisations to compete against NHS owned providers (which, for the avoidance of doubt, still include FTs). This encouragement of diversity of provision is part of the attempt to increase competition among providers, which is a key economic mechanism in markets.

The economic goal of increasing autonomy for providers of care has been promoted in several ways. The Coalition policy is that all NHS trusts should become FTs by 2014 [63], which is coupled with statements that there will be a significant reduction in command and control activity from the centre (for example, the use of targets is to be abolished [64] – although the new outcomes framework [65] seems remarkably similar to a target in practice). FTs will also be able to increase the amount of income they earn from private patients above the existing cap (the amount prior to elevation to FT status) set in previous legislation (HCSA s 165). FTs will now be able to earn anything up to half of their income from private sources, provided this does not interfere with the discharge of their primary functions to treat NHS funded patients (HCSA s 164). In the context of increasing financial stringency in the NHS budget, it will be important to investigate the extent to which this provision allows privately funded care to substitute for care previously funded by the NHS. If it does, some of the demand side of the healthcare market will have become closer to classical economic market principles, where consumers make and fund their own consumption decisions. Other goals, particularly concerning fairness of access to care, may well be compromised, however.

In sum, the HSCA contains attempts to increase the force of economic market mechanisms in the NHS quasi market, mainly in respect of provider regulation and autonomy, but these efforts have been attenuated by the provisions aimed at ensuring that other important goals of the NHS are still taken into account.

## Conclusion

Having surveyed the theory of markets, the nature of healthcare and the available evidence concerning the English quasi-market in the NHS, two points are evident. It is not possible to construct a market conforming to classical economic principles in respect of healthcare. Moreover, it is not desirable to do so, as goals (such as fairness of access to care) are also crucial, and these are not goals which markets can deliver.

I now turn to the advantages of a using the organisational structures of a hierarchy for the delivery of healthcare in the English NHS. *Firstly*, in the English political context, where central government will inevitably be held politically responsible for health services, the hierarchical structure may give the government the best opportunity to exercise control over the NHS. Unlike the quasi-market (where, in principle, organisational separation and contracting precluded the use of direct sanctions against provider Trust managers) in a true hierarchy, sanctions are available to higher echelons to use against all lower levels. This can improve accountability. *Secondly*, a hierarchy allows strategic planning and allocatively efficient decisions to take place at the appropriate level of aggregation. *Thirdly*, institutional economic analysis indicates that hierarchical governance structures may well be most efficient, in terms of reduction of transactions costs, for complex services. *Fourthly*, the authority relationships contained in a hierarchy can be used to foster important normative influences, such as public service ethics, while any confusion in public servants’ minds about the role of competition and self interest which could be caused by the introduction of market mechanisms [66] will be absent.

Whatever governance structures are used, there will always be problems associated with systems of planning and delivering public services. It is arguable that in the context of British political culture, a public hierarchy is the least bad solution. As Jackson [67] points out:

‘*Given that many activities*, *which were organised through the public sector*, *were located there because of the failure of markets to allocate them effectively and given our understanding of what markets cannot do*, *then it is a bit strange to believe that the problems of bureaucracy could be solved by taking these services out of the traditional bureaucracy and confronting them with greater amounts of competition and managerial control*.’ (p 13)

## Competing interests

None.

## Declarations

Publication of this supplement was funded by the Nuffield Trust.

This article has been published as part of *BMC Health Services Research* Volume 13 Supplement 1, 2013: The Limits of Market-based Reforms. The full contents of the supplement are available online at http://www.biomedcentral.com/bmchealthservres/supplements/13/S1.
